# Oxide Coating Role on the Bulk Structural Stability of Active LiMn_2_O_4_ Cathodes

**DOI:** 10.1021/acs.jpcc.3c00342

**Published:** 2023-05-03

**Authors:** Francesco Paparoni, Emin Mijit, Hamideh Darjazi, Francesco Nobili, Andrea Zitolo, Andrea Di Cicco, Rahul Parmar, Roberto Gunnella, S. Javad Rezvani

**Affiliations:** † Sez. Fisica, Scuola di Scienze e Tecnologie, Universitá di Camerino, via Madonna delle Carceri, I-62032 Camerino, Italy; ‡ Sez. Chimica, Scuola di Scienze e Tecnologie, Universitá di Camerino, via Madonna delle Carceri, I-62032 Camerino, Italy; § Synchrotron SOLEIL, L’Orme des Merisiers, Départementale 128, 91190 Saint-Aubin, France

## Abstract

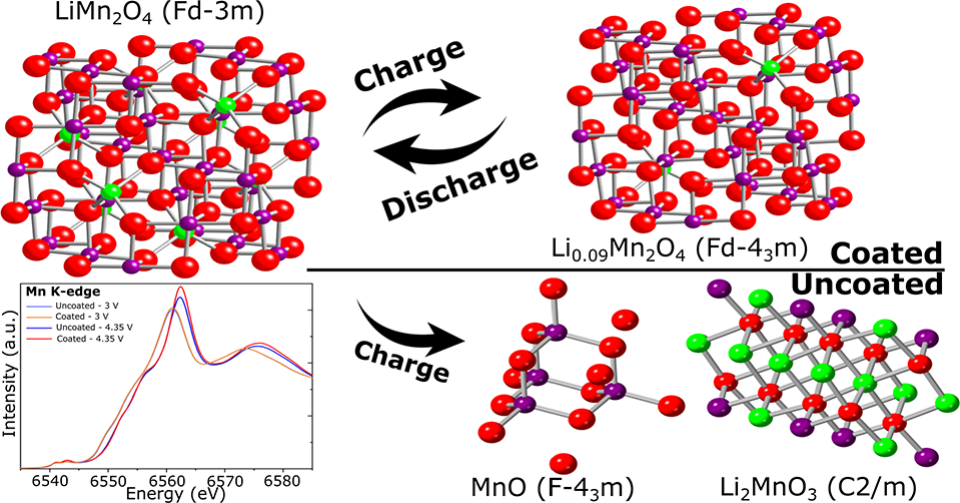

The protective coating of the electrode materials is a known source of improvement of the cycling performances in battery devices. In the case of the LiMn_2_O_4_ cathodes, the coating with a thin alumina layer has been proven to show performance efficiency. However, the precise mechanism of its effect on the performance improvement of the electrodes is still not clear. In this work we investigate alumina-coating-induced effects on the structural dynamics of the active materials in correlation to the modified solid electrolyte interface dynamics. The local structures of coated and uncoated samples at different galvanostatic points are studied by both soft X-ray absorption measurements at the Mn L-edges and O K-edge (in total electron yield mode) and hard X-ray absorption at the Mn K-edge (in transmission mode). The different probing depths of the employed techniques allowed us to study the structural dynamics both at the surface and within the bulk of the active material. We demonstrate that the coating successfully hinders the Mn^3+^ disproportionation and, hence, the degradation of the active material. Side products (layered Li_2_MnO_3_ and MnO) and changes in the local crystal symmetry with formation of Li_2_Mn_2_O_4_ are observed in uncoated electrodes. The role of alumina coating on the stability of the passivation layer and its consequent effect on the structural stability of the bulk active materials is discussed.

## Introduction

Li-ion batteries have been among the most used energy storage devices for over a decade. They are one of the first choices as a power supply for consumer electronics and electric vehicles.^1^ Today, with respect to the high capacity obtainable from anode materials, the limited capacity of the cathode materials represents the bottleneck of the technological advancement for these battery devices.^2,3^ Recent studies highlighted the critical role of the active material–electrolyte interface in the prevention of the capacity fading of the cathode.^4,5^ The repeated cycling of the battery electrodes outside the electrolyte’s voltage window of stability provokes the formation of a protective passivating layer on the electrodes, the so-called solid electrolyte interphase (SEI) on the anodes and cathode–electrolyte interphase (CEI) on the cathode.^6,7^ Such protective layers prevent ulterior oxidation/reduction at the active material–electrolyte interface and stabilize the diffusion of Li^+^ ions, essential for battery operation. It is known that in the presence of the electrolyte salt the redox process starts almost immediately, while the formation of passivation layers occurs primarily during the first cycle of battery operation.^7−9^ However, a limited growth of the passivation layers on Li-ion cathode materials can result in a consequent aging of the electrodes and substantial deterioration of the active material. Thus, one of the primary goals of the research on cathode materials is to improve the interfacial process of the reversible ion uptake, while limiting the deterioration of the active material during cycling.^9−12^ A leading process for the enhancement of interface stability and diffusion has been the introduction of a second oxide coating on the active materials. These kinds of coatings are mostly insulating metal oxides that are expected to maintain the ionic diffusion into the active materials while hindering the interfacial deteriorations. While several studies have indicated performance improvement by the secondary oxide coating of the active materials, particularly at the interface, the bulk structural dynamics of the active materials in the presence of such coatings remain unclear.

Lithium manganates^13^ are one of the classes of cathode active materials that have shown superior properties compared with their similar counterparts.^13−17^ In particular, LiMn_2_O_4_ (LMO) is interesting for the low cost and high working potential. Nevertheless, LMO’s thermal and compositional stability must be the object of further studies, which could result in improved stability and efficiency of the cathode material without resorting to Ni or Co doping, detrimental for safety and economical issues, respectively. Stoichiometric LiMn_2_O_4_ has a spinel structure (space group *Fd*3̅*m*), characterized by the general formula A[B_2_]O_4_, with A divalent ions in tetrahedral symmetry (*T*_*d*_) and B trivalent ions in octahedral symmetry (*D*_3*d*_). In particular, Li^+^ ions occupy the 8*a* tetrahedral site and Mn^3+^ or Mn^4+^ the 16*d* octahedral site. Oxygen anions occupy the 32*e* site in the cubic close-packed *C*_3*v*_ symmetry.^18,19^ However, the LMO spinel crystal structure has shown to be unstable during cycling, with a change of the crystal symmetry from cubic to tetragonal due to Jahn–Teller (J–T) distortion in octahedrally coordinated Mn^3+^, which results in a volumetric expansion.^20,21^ Moreover, the Mn^3+^ disproportion (2Mn^3+^ → Mn^2+^ + Mn^4+^) triggers the dissolution of Mn^2+^ species, which are leached into the electrolyte. Such Mn dissolution is one of the main effects associated with the capacity fading of LMO cathode materials and, hence, needs to be minimized.

Recently it has been shown that the oxide coating (e.g., alumina) of the LMO active material reduces the severe decomposition at the active material–electrolyte interface, resulting in the formation of a more stable CEI.^21^ However, the bulk propagation of these interfacial interactions and their effect on the bulk structure of the active material remain unknown. In this work we have exploited X-ray absorption spectroscopy (XAS) to investigate the effect of Al_2_O_3_ coating on the disproportion mechanism within LiMn_2_O_4_ electrodes and study the correlation with the Jahn–Teller distortions and side products suppression within both coated and uncoated bulk structures. The XAS measurements were performed in two soft and one hard X-ray energy ranges in order to compare interface and bulk dynamics at two different galvanostatic points.^22,23^ The results are organized in two parts. The first part focusses on the soft XAS analysis with which it is shown how the Mn^3+^ disproportion is hindered via coating, resulting in a reduced Mn^2+^ dissolution at the electrode/electrolyte interface. In the second part, the preservation of the structural order within the bulk structure of the coated samples is investigated using hard X-ray absorption near-edge structure (XANES) and extended X-ray absorption fine structure (EXAFS) analysis. The improvement of the battery performance based on the consequent bulk structure dynamics and side product formation is discussed in detail.

## Experiment

Al_2_O_3_–LiMn_2_O_4_ synthesis and the electrode preparation process followed the solid-state method described in refs (14 and 24). The method consisted of milling and calcination of Li_2_CO_3_ at 400 °C for 10 h, followed by 48 h of reground and calcination at 750 °C of MnCO_3_. A few nanometers of Al_2_O_3_ coating (3 wt %) was obtained via the coprecipitation route method.^25^ The LMO powder, immersed in a 0.02 M solution of Al(NO_3_)_3_·9H_2_O and deionized water, was stirred while adding ammonia solution in order to obtain Al(OH)_3_ in precipitation. The slurry, separated and washed with deionized water, was dried in a vacuum at 40 °C. After calcination at 450 °C with 10 °C/min heating and cooling rate for 120 min in air, Al_2_O_3_–LiMn_2_O_4_ is finally obtained. To prepare cathode slurries, the active material was blended with sodium carboxymethyl cellulose (Na-CMC) and SuperC65 conductive carbon (80:10:10 mass ratio) in deionized water. The slurry was then cast onto Al foil collector using the doctor blade technique, making a 200 μm thick laminate, and then dried at 80 °C for 3 h. Before transferring it to a glovebox, the laminate was pressed, and a second drying step was performed at 120 °C under vacuum overnight. The active material within the electrode was 2 mg cm^–2^. Lithium metal was employed as a counter and reference electrode, separated by glass fiber (Whatman GF/A), in 1 M LiPF_6_ in EC:DMC (1:1 v/v) electrolyte.

LiMn_2_O_4_ uncoated samples as well as Al_2_O_3_-coated ones were prepared in two conditions: fully charged electrodes at 4.35 V; fully discharged electrodes after the first cycle, at 3 V. The charge steps were applied at constant current (1 C rate, 1 C assumed to be 148 mAg^–1^ with respect to the active material mass), followed by a constant-potential equilibration step, in which the current was left to decay below C/20 (i.e., *I* = 7.5 mA g^–1^). All potentials are given respect to the Li^+^/Li redox couple. For X-ray absorption spectroscopy (XAS), electrodes were dried in an Ar atmosphere and protected in sealed packs while moved to the measurement chambers. Then, via an argon-filled load lock chamber, samples were placed in the experimental chambers. Soft XAS measurements were performed in total electron yield mode (TEY), using the radiation at the exit of the 8.1 bending magnet of the ELETTRA synchrotron facility in Trieste (Italy) (BEAR end-station BL8.1L). The incident light was horizontally polarized, fixing the incidence angle of the light with respect to the sample surface at 45° with the s polarization. The spectral energy was calibrated with respect to the carbon π–π* transitions. The data were normalized to the incident photon flux.^23^ Hard XAS experiments at the Mn K-edge were performed in transmission mode on equivalent samples at the bending-magnet beamline SAMBA in SOLEIL synchrotron. Nine acquisitions were collected for each sample. Each acquisition was normalized to the incident photon flux and calibrated by aligning the first maximum of the first derivative of the reference spectra, acquired from a Mn foil, to the theoretical value of the Mn K-edge absorption threshold (6539 eV).^26^ The spectra were then merged with the software ATHENA.^27^ EXAFS analysis was performed using the GNXAS package.^28^

## Results

**Figure 1:**
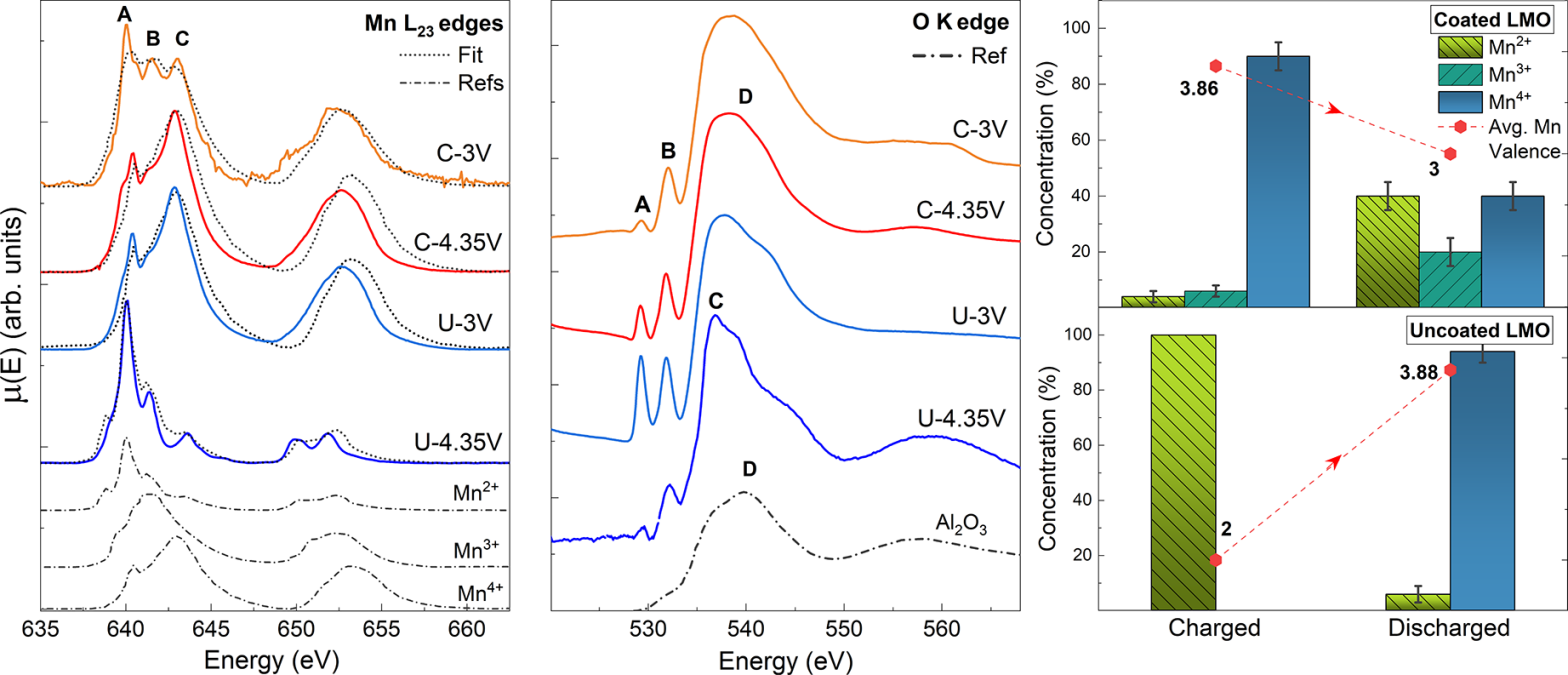
Soft XAS spectra of coated (C) and uncoated (U) samples in fully charged (4.35 V) and discharged conditions (3 V) at the Mn L_2,3_ and oxygen K-edges. At the bottom of the figures, the reference spectra of manganese oxides^29^ and alumina^30^ are plotted with an intensity divided by a factor of 2. The best fits of the Mn L-edge spectra are displayed as dashed lines and confronted with the experimental data (continuous lines). The results of the fits are highlighted in the bar graph (right), with the respective error bars. The average oxidation states of the Mn ions at the two galvanostatic points are indicated by the label of the red points.

The XAS spectra of Al_2_O_3_ coated and uncoated LiMn_2_O_4_ cathodes taken at two galvanostatic points by soft X-ray total electron yield (TEY) measurements are reported in Figure 1. TEY measurements with a mean probing depth between 2 and 10 nm^21^ provide surface information and the structure at the electrode/electrolyte interface. At the Mn L_23_ edge, the spectra show three distinct peaks at 640.0 eV (A), 641.5 eV (B), and 643.0 eV (C) which can be assigned to the Mn^2+^, Mn^3+^, and Mn^4+^ states, respectively.

At 4.35 V the coated sample shows a high Mn^4+^ concentration, as expected in the delithiation half-cycle, while Mn^2+^ dominates the spectra in the uncoated case. After the discharging process, the coated sample shows higher intensities of the peaks related to lower oxidation states (A and B). Conversely, the average oxidation state of uncoated samples drastically increases to Mn^4+^. The qualitative assessments were confirmed by performing a linear combination fit (LCF) of the experimental data using suitable manganese oxide references (see Figure 1 and the Supporting Information). As shown by the bar graph, at 4.35 V the oxidation state of the superficial Mn ions within the coated sample is close to 4+, with a minimum trace of Mn^3+^ (6%) and Mn^2+^ (4%) species. The best fit obtained for the uncoated sample at 4.35 V points to the complete reduction of the active material to Mn^2+^. After the discharge, the increase of the Mn^2+^ and Mn^3+^ concentrations within the coated sample results in the expected reduction of the average Mn oxidation state. On the other hand, the drastic increase of the oxidation state in the uncoated case is proven by the decrease of the Mn^2+^ content (6%) and concurrent increase of the Mn^4+^ concentration. These results are in good agreement with previous work on similar samples.^21^

A similar process is also observed in the O K-edge spectra. The alumina coating on the coated samples is pinpointed by a broad feature at ∼540 eV (D), resulting from the Al–O contribution (see reference Al_2_O_3_ spectra in Figure 1). All samples show two components at 529 and 531 eV (peaks A and B), attributed to the Mn–O phases in the active material and to the hybridization of O 2p and Mn 3d electrons from the e_g_ state.^31^ The increase of the Mn oxidation state by charging is confirmed in the O K-edge spectra of coated samples by the raising of component A. The higher Mn valency of the coated sample at 4.35 V compared with the stoichiometric LiMn_2_O_4_ is expected via the removal of Li ions from the structure.^21^ These results are in agreement with the preservation of the initial cubic symmetry of LiMn_2_O_4_ and further formation of spinel λ-MnO_2_.^5,32^ The uncoated sample shows an opposite behavior, in agreement with the manganese L-edge results. The decrease of the Mn valence upon charging is confirmed by the quenching of component A as well as by the increase of the component at 537 eV (peak C), indicating the formation of Mn^2+^ species.^29^ The distinct dynamics at the surface of the uncoated sample indicate the presence of an unstable interface layer at which the disproportionation leaves a high trace of Mn^2+^. The dissolution of Mn^2+^ ions results in a drastic increase of the superficial oxidation state at 3 V, highlighted at the O K-edge of uncoated samples by the drastic raising of component A and quenching of C.

**Figure 2:**
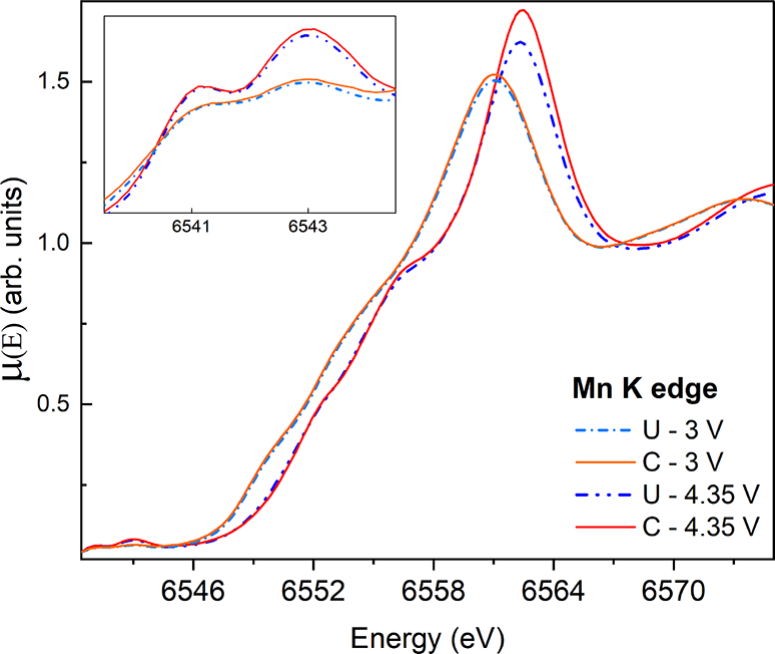
XANES spectra, acquired at the Mn K-edge, of coated (C) and uncoated (U) samples at 3 and 4.35 V. Uncoated samples are represented by dashed lines. Inset: the pre-edge peaks.

**Figure 3:**
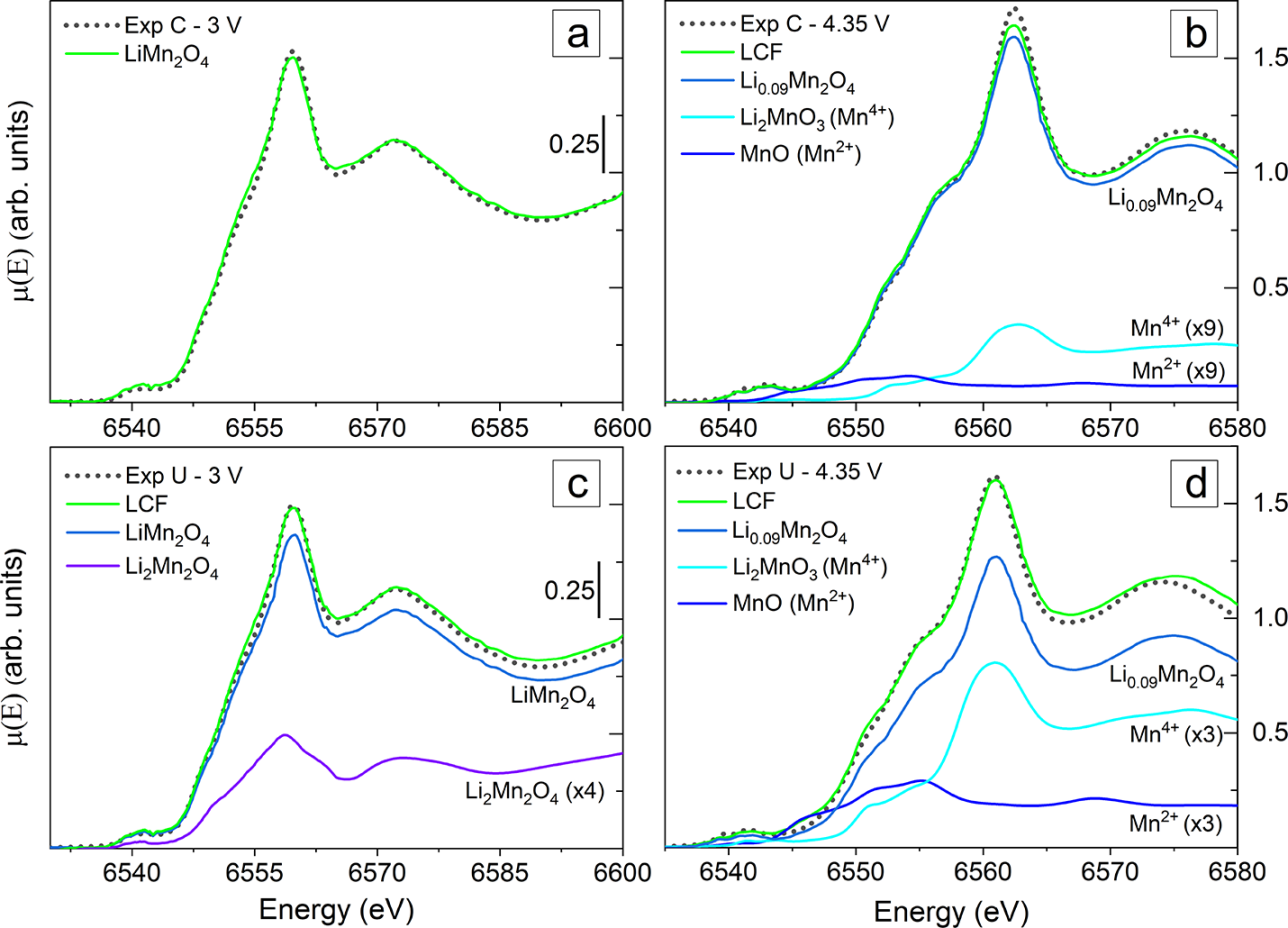
(a) Comparison of the coated sample at 3 V with a LiMn_2_O_4_ reference.^22^ (b) LCF of the coated sample at 4.35 V with delithiated LMO, MnO, and layered Li_2_MnO_3_ references.^33,35^ (c) LCF of the discharged uncoated sample with cubic LiMn_2_O_4_ and tetragonal Li_2_Mn_2_O_4_ references.^22,36^ (d) LCF of the uncoated sample at 4.35 V with the delithiated LMO, MnO, and Li_2_MnO_3_ references.^33,35^ To improve visualization, the minor components have been multiplied by the factors indicated.

The propagation of the disproportionation and redox, originating at the interface, into the bulk of the active material was investigated using hard X-ray absorption spectroscopy at the Mn K-edge. Figure 2 shows the X-ray absorption near-edge structure (XANES) spectra of coated and uncoated samples at 3 and 4.35 V. Increasing the charging potential results in a shift of the white line from 6561.1 to 6562.4 eV (in uncoated samples) and 6561.1 to 6562.5 eV (in coated samples). The relative intensities of the white lines and pre-edge peaks (inset in Figure 2) are also higher by the increase of the charging potential. These modifications can be attributed to the increase of the Mn oxidation state and overall structural order within the samples.^33,34^ The white line fluctuations can be correlated to the deintercalation of lithium ions from the structural matrix (i.e., *x* in Li_*x*_Mn_2_O_4_), resulting in the oxidation of the Mn. The shift of the white line and the Mn valence state are inversely proportional to *x*. If no side products are formed, a complete deintercalation of Li ions (*x* = 0) leads to the formation of spinel λ-MnO_2_. Our results, in comparison with ref (34), suggest a Li content of *x* = 1 at 3 V and 0.25 > *x* > 0.08 at 4.35 V. In a previous work on similar samples,^21^ we observed via Raman spectroscopy the formation of a defective layered structure in the uncoated sample. Here, the relatively lower white line intensities displayed by the uncoated samples suggest a lower structural order with respect to the coated case.^22^ Considering the possible side products formed as a result of the disproportionation mechanism and J–T distortion (discussed later), a LCF of the XANES spectra at different stages was performed using suitable references (see Figure 3 and the Supporting Information).

**Figure 4:**
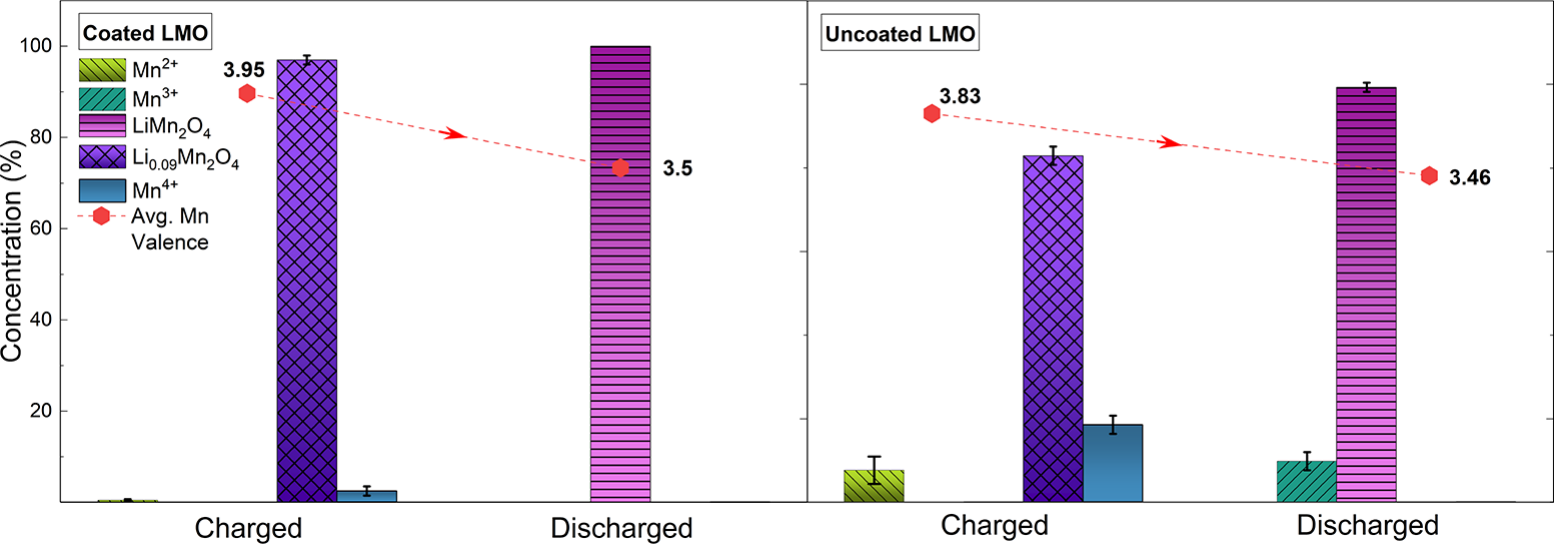
Species content (%) in coated and uncoated samples, obtained via LCF with reference XANES spectra as shown in Figure 3. In red is shown the resulting average of the Mn oxidation state.

The results of the best fits are summarized in Figure 4 (see also Supporting Information). The coated sample at 3 V shows the initial LiMn_2_O_4_ structure (see Figure 3a). At 4.35 V, this sample fits well with a delithiated Li_0.09_Mn_2_O_4_ reference (Figure 3b), in agreement with the expected Li removal for potential over 4 V,^33^ in addition to a layered Li_2_MnO_3_ and a negligible amount of the MnO. On the other hand, the discharged uncoated sample (3 V) shows a cubic LiMn_2_O_4_ structure along with a tetragonal Li_2_Mn_2_O_4_ component (see Figure 3c). This sample at 4.35 V shows the expected Li_0.09_Mn_2_O_4_ structure resulting from the Li ions removal, along with a higher content of layered Li_2_MnO_3_ and MnO with respect to the coated sample (see Figure 3d). The higher ratio of these side products in uncoated samples confirms a lower structural order in these samples,^22^ justifying the lower white line intensity in the XAS spectra of these samples as well (see the Supporting Information for further details).

**Figure 5:**
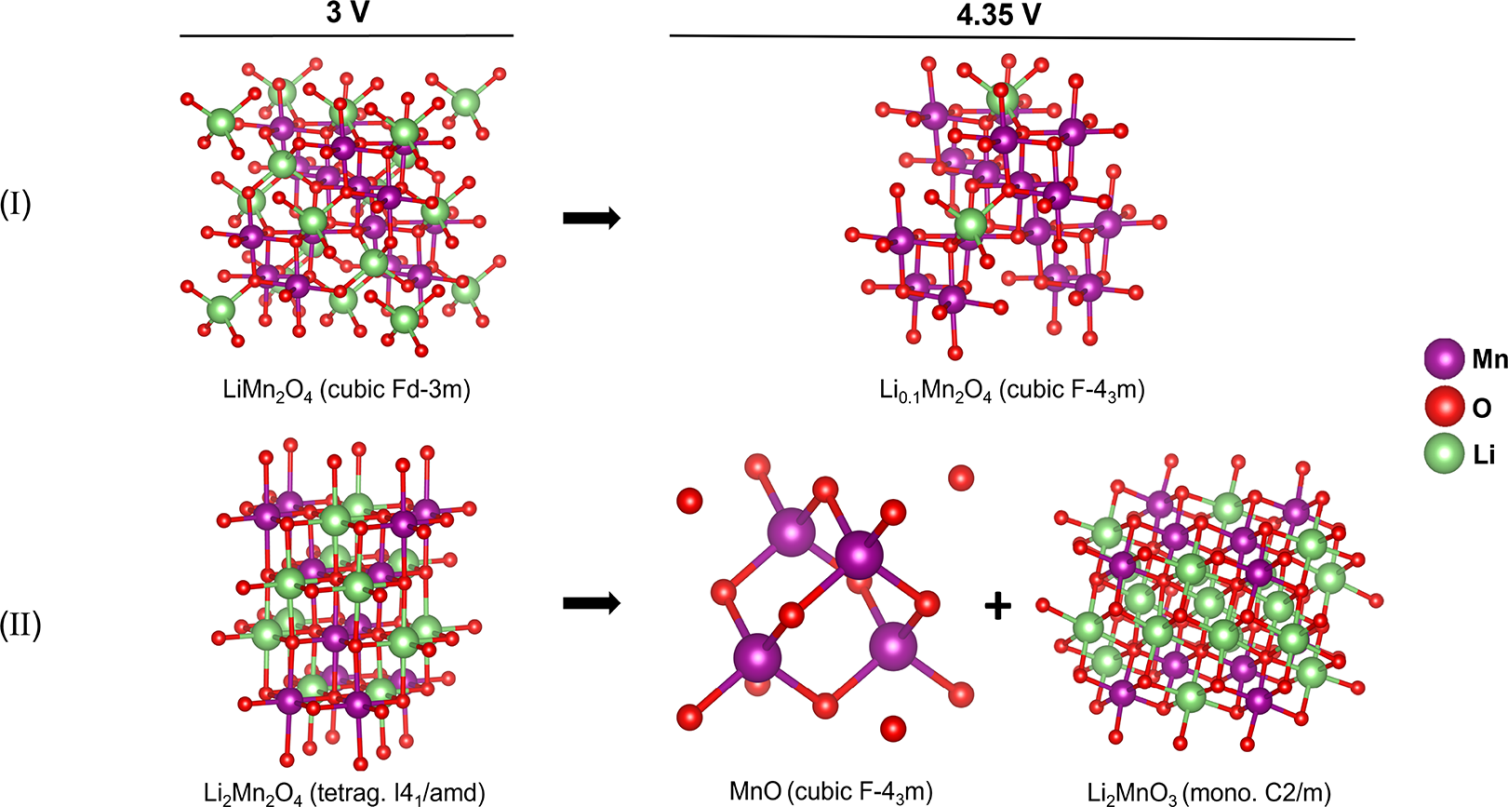
Proposed models of the LMO at 3 and 4.35 V: (I) the expected lithium intercalation that preserves the cubic symmetry; (II) disproportion of defective phase with Li in octahedral sites formed by J–T distortion.

The suggested structural dynamics were also examined via extended X-ray absorption fine structure (EXAFS) analysis. The fit was performed using γ^(2)^ XAS two-body signals, relative to the two-body distribution functions *g*_2_(28) associated with different Mn–O and Mn–Mn bonds within several coordination shells. The γ^(2)^ signals were calculated from suggested reference structures (see the Supporting Information). A suitable background was realized with a linear function for the pre-edge region and fifth-order spline functions for the post-edge region. Around 6629 eV a second edge contribution, due to the double-electron excitation channel related to a KM shake-off (1s → ϵp, 3s → ϵs), was included as well in the background function.^37^ This feature is located 90 eV above the Mn K-edge, near the Fe 3s ionization energy (92 eV), in good agreement with the *Z* + 1 approximation.

The first refinement was performed on the coated sample at 3 V, being the closest structure to that of the original spinel structure. A cubic spinel LiMn_2_O_4_ structure (cubic, *Fd*3̅*m* space group) was used as the reference. Fitting was performed, fixing the coordination numbers (CN) to that of the reference values and varying distances (*R*) and mean-square fluctuations of the interatomic distances (i.e., Debye–Waller factor, σ^2^).

**Figure 6:**
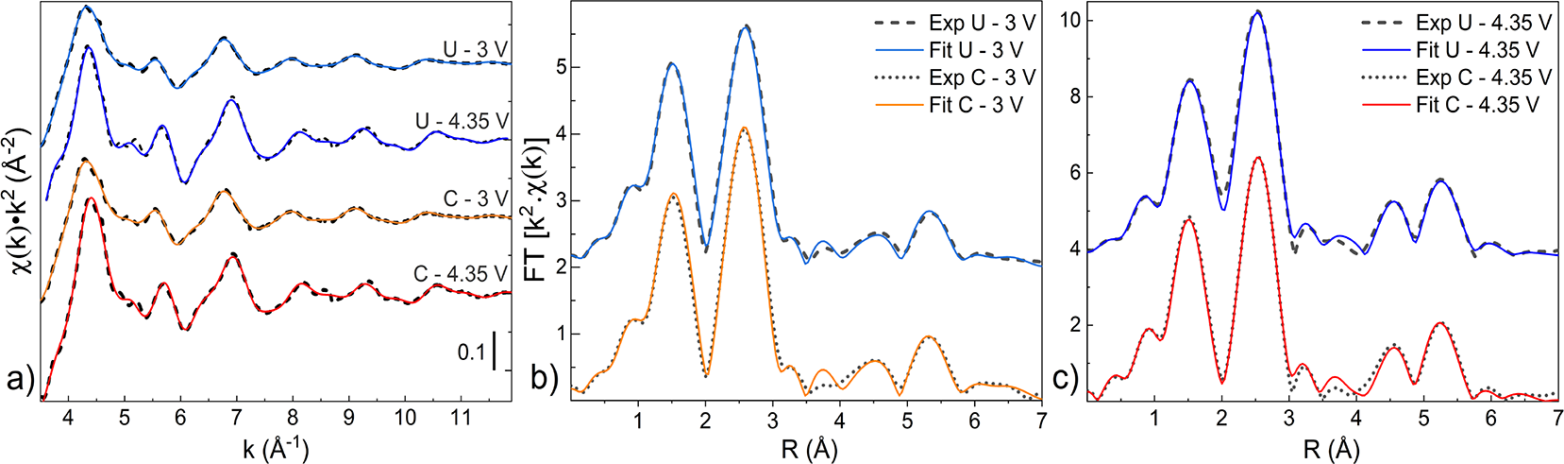
*K*^2^-weighted EXAFS spectra (a) and corresponding Fourier transform of coated (C) and uncoated (U) samples discharged (b) and fully charged (c). The experimental data are plotted as dashed lines, together with the fit (continuous lines).

The best fit was realized with 8 two-body components with an excellent agreement with the experimental data using simple Gaussian approximations (see Table 1 and the Supporting Information). The atomic distances in the first two shells (1.907 and 2.915 Å) are in excellent agreement with the spinel LiMn_2_O_4_ structure as observed also in refs (22 and 33). The low Debye–Waller factors confirm the high structural order in this sample. At 4.35 V, using a similar fitting procedure, a reduction of the Debye–Waller factors related to the 1st, 2nd, 4th, and 6th shell was observed. On the other hand, as reported in Table 1, the 3rd, 5th, 7th, and 8th are related to weak scattering peaks, and hence, the σ^2^ (Å) fluctuations observed are comprised within the large computational error. This reduction, along with the intensity increase of the main scattering peaks (see Figure 6b,c and the Supporting Information), indicates an improvement of the overall structural order which is expected by the lithium extraction and formation of the Li_*x*_Mn_2_O_4_.^33^ This is also confirmed by the Mn–Mn bond distance reduction (2.915 to 2.858 Å) within the second shell due to the increase of the Mn oxidation state.^33^ The addition of further side components such as MnO and Li_2_MnO_3_ did not improve the fitting results due to their negligible content in this sample.

**Table 1:**
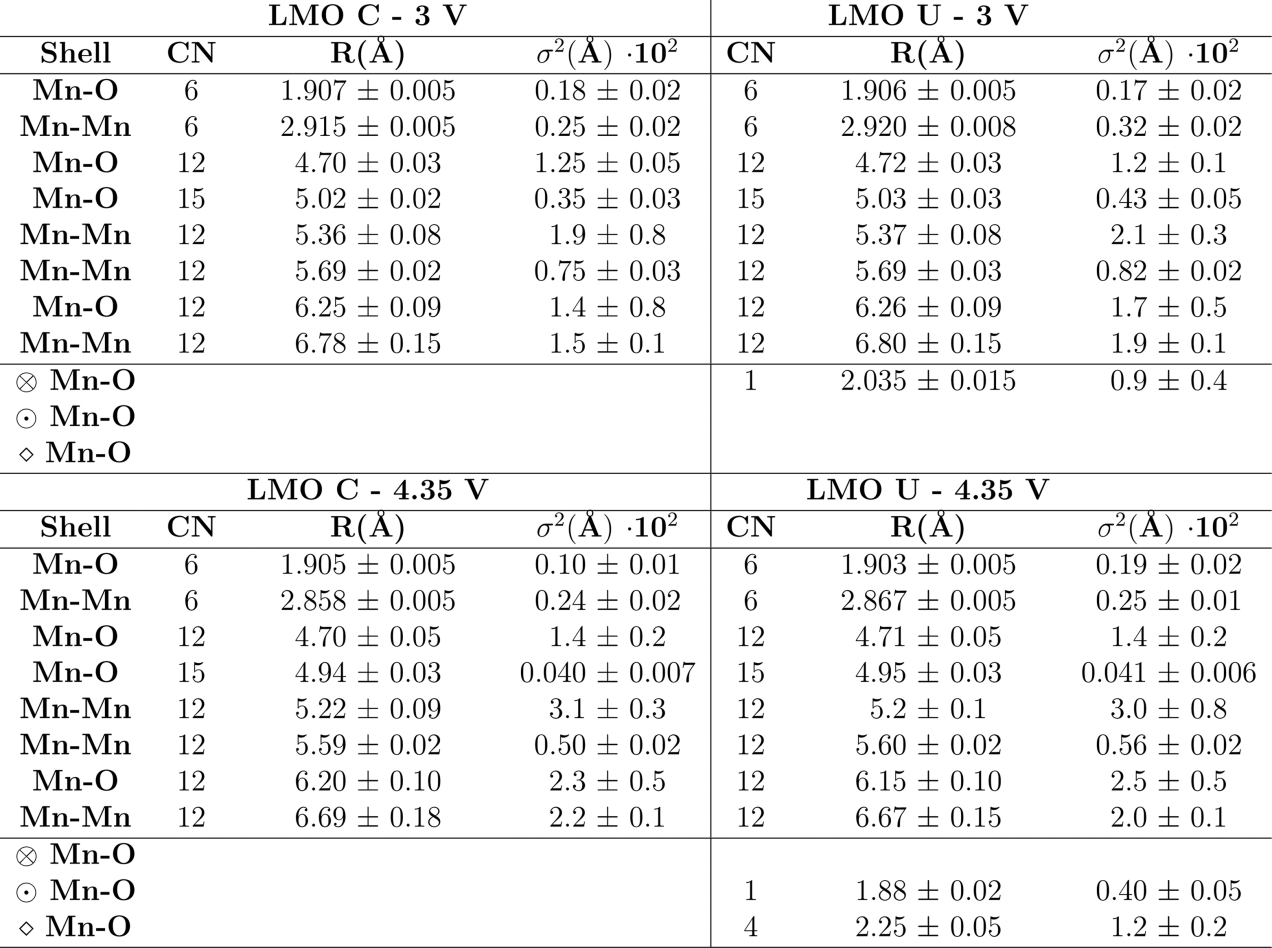
Best-Fit Values of the EXAFS Spectra^a^

a Debye–Waller factors (σ^2^) have been multiplied by a 10^2^ factor to improve visualization. In the last three rows are reported the first-shell contributions relative to the Mn–O bond of Li_2_Mn_2_O_4_ (⊗), Li_2_MnO_3_ (⊙), and MnO (◇).

The uncoated sample at 3 V, however, does not comply with the spinel structure used for the fitting of the coated samples. The best fit was achieved including the first Mn–O bond of the Li_2_Mn_2_O_4_ into the overall structure, in agreement with the XANES results. The higher Debye–Waller factors associated with the main scattering peaks (2nd, 4th, and 6th shell in Table 1), with respect to the coated case, can be attributed to the J–T distortion. This result confirms the lower structural order within this sample. In particular, the relatively lower intensity of the first Mn–Mn bond component is in agreement with the formation of Li_2_Mn_2_O_4_^22,36^ (see Figure 6b and the Supporting Information) which suggests a partial change of the crystal symmetry from cubic to tetragonal, as expected from the J–T distortions. At 4.35 V, the best fit for the uncoated sample was obtained by the addition of the first shells of Li_2_MnO_3_ and MnO. The increase of the scattering peaks intensity, as well as the shortening of the 2nd shell Mn–Mn bond, confirms the oxidation of the Mn due to Li extraction. However, the decrease of the first scattering peak intensity and the broadening around 2 Å (see Figure 6c and the Supporting Information) correlate with the formation of Li_2_MnO_3_ and MnO side products, in agreement with the XANES results. The best result was obtained using a coordination number of 4 for the MnO, suggesting that in this side product the Mn atoms are allocated in tetrahedral sites.

## Discussion and Conclusions

Our results suggest that the oxide coating of the LMO active material induces the formation of a more stable interface with the electrolyte that reduces the Mn^2+^ dissolution due to the Mn^3+^ disproportionation, preserving the expected redox cycle required by the battery operation. Comparison of our LCF of the soft X-ray TEY and hard X-ray transmission mode measurements analysis shows similar superficial and bulk active material dynamics in the alumina-coated samples. Mn ions are oxidized to Mn^4+^ both in bulk and at the interface, as a consequence to Li^+^ extraction, while the other Mn valences (indicating formation of the side products) are negligible in bulk compared with the electrolyte interface. Upon discharge, the Mn^4+^ ions at the interface are reduced to Mn^3+^ and Mn^2+^, while the bulk structure goes back to the initial LiMn_2_O_4_ structure, suggesting the segregation of the Mn^2+^ species mainly at the electrode/electrolyte interface. On the other hand, in charged uncoated samples, the interfacial Mn ions are completely reduced to Mn^2+^, while within the bulk the disproportion of Mn^3+^ appears more evident. Together with the expected delithiated phase, the bulk structure analysis highlights the presence of several side products (e.g., 7% component of MnO and 17% of Li_2_MnO_3_). Upon discharge, at the interface, the Mn^2+^ species are exchanged by Mn^4+^ ions while the spinel bulk structure coexists with the reduced Mn^3+^ species with tetragonal symmetry.

In the noncoated sample, the aggressive deterioration of the active material results in a significant inhomogeneity of the lithium distribution, with the formation of several side products such as the tetragonal Li-rich phase (Li_2_Mn_2_O_4_), MnO, and layered Li_2_MnO_3_ at distinct charging stages. The formation of the Li_2_Mn_2_O_4_, resulting from a nonuniform lithium distribution within the bulk structure of the uncoated sample, can be explained by J–T distortion. In such a case, during the intercalation Li ions can occupy octahedral sites instead of tetrahedral, favoring the local increase of lithium concentration.^38^ This transition is accompanied by the change of the crystal symmetry from cubic to tetragonal. J–T distortion in LMO cathodes is strongly dependent on the J–T active Mn^3+^ ions content. The structural deterioration that occurs in the absence of the alumina coating can be explained by a high concentration of the J–T active Mn^3+^ in these samples. In the uncoated material, the charge produced by the oxidation of the electrolyte can be transferred to the Mn^4+^ ions, which reduce to Mn^3+^, increasing the concentration of J–T active sites on the sample surface.^39^ On the other hand, the alumina coating can suppress the Mn^3+^ formation at the electrode–electrolyte interface. The Mn 3 states coupling with the 2p orbitals of the oxygen atoms of the alumina coating favors the oxidation of the Mn^3+^ to a non-J–T active Mn^4+^.^40^ Hence, the suppression of the J–T active ions inhibits the cubic-to-tetragonal transition, preventing the formation of Li_2_Mn_2_O_4_ in the coated sample.

During the lithium extraction, the oxidation of the electrolyte and the reduction of Mn^4+^ to Mn^3+^ result also in oxygen loss. As described by Ben et al.,^41^ the oxygen loss at the surface of the LMO cathode allows octahedrally coordinated Mn ions to migrate to the now empty Li tetrahedral sites. The higher mobility of Mn^3+^ in the spinel lattice results in the formation of the J–T distorted structure, such as Li_2_Mn_2_O_4_. At higher potentials, the defective spinel decomposes due to the Mn^3+^ disproportion. The Mn^2+^ can remain in the Li tetrahedral sites, forming MnO, while Mn^4+^ moves to octahedral sites, forming layered Li_2_MnO_3_, energetically favorable with respect to the delithiated spinel structure Li_*x*_MnO_2_.^41^ This disproportion mechanism can be described by the following equation:
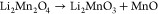
1This reaction is also enhanced by the dissolution of the Mn^2+^ and the consequent increase of the Li/Mn and O/Mn ratios, which promotes a transition from spinel LiMn_2_O_4_ to layered Li_2_Mn_2_O_4_ or Li_2_MnO_3_ phases, forming heterostructure within the initial cubic spinel structure.^42^ Higher charging potentials favor further formation of the layered Li_2_MnO_3_, which is expected to leave regions with weakly bounded Mn atoms, favoring the delamination of the surface layer into the electrolyte and, hence, aggravating the Mn dissolution.^1,41^ The higher concentration of the Li_2_MnO_3_, compared with the MnO, is due to the solubility of the Mn^2+^ species in the electrolyte, in agreement with previous work.^5^

In conclusion, the oxide coating mitigates the Mn^2+^ dissolution into the electrolyte and hinders the significant distortions, otherwise propagated through the bulk structure. This shielding effect reduces the loss of active material, while also preventing the formation of side products that can reduce the mobility of the Li ions. These effects lead to improved cyclability and final performance of the electrode.

## Supporting Information Available

Linear combination fits results (Tables S1 and S2); supplementary LCF (Figure S1); comparison of the FT of the EXAFS signals with side products first shell signal (Figure S2); structure diagram of the Li extraction (Figure S3) (PDF)
