# Supplementary material for: Oxide Coating Role on the Bulk Structural Stability of Active LiMn2O4 Cathodes
Source: J Phys Chem C Nanomater Interfaces. 2023 May 3;127(18):8649–56. doi: 10.1021/acs.jpcc.3c00342 (PMC10184565; doi:10.1021/acs.jpcc.3c00342)
Supplement: Supplementary file 1 — jp3c00342_si_001.pdf [file jp3c00342_si_001.pdf]

Oxide Coating Role on the Bulk Structural Stability of Active $LiMn_2O_4$ Cathodes - Supporting Information –

F. Paparoni,[1,3] E. Mijit,[1] H. Darjazi,[2] F. Nobili,[2] A. Zitolo,[3] A. Di Cicco,[1] R. Parmar,[1,4] R. Gunnella,[1] and S. J. Rezvani[1,5,*]

[1]Sez. Fisica, Scuola di Scienze e Tecnologie, Università di Camerino, via Madonna delle Carceri, I-62032 Camerino, Italy
[2]Sez. Chimica, Scuola di Scienze e Tecnologie, Università di Camerino, via Madonna delle Carceri, I-62032 Camerino, Italy
[3]Synchrotron SOLEIL, L'Orme des Merisiers, Départementale 128, 91190 Saint-Aubin, France
[4]Elettra - Sincrotrone Trieste, S.C.p.A., SS14 – km 163.5 in Area Science Park, 34149, Trieste, Italy
[5]CNR-IOM, Bazovizza, Trieste, Italy

1. Linear Combination Fit.

Linear combination fit (LCF) on the TEY measurement acquired at the Mn L_{23} edges on equivalent samples were carried out in a previous work[1] using simulated XAS references. In this work, the LCF have been carried out with Mn^{2+} (MnO), Mn^{3+} (Mn_2O_3) and Mn^{4+} (MnO_2) experimental references[11]. Our results are in good agreement with our previous work and are reported in Tab.S1 (a).

a) Total Electron Yield (TEY) Mn L edges

Sample charge state	Coated 2+	Coated 3+	Coated 4+	Coated Avg. valency	Uncoated 2+	Uncoated 3+	Uncoated 4+	Uncoated Avg. valency
4.35 V	4 ± 2	6 ± 2	90 ± 1	3.86 ± 0.14	93 ± 3	4 ± 2	3 ± 1	2.1 ± 0.16
3 V	40 ± 5	20 ± 2	40 ± 1	3.1 ± 0.2	6 ± 3	0	94 ± 3	3.9 ± 0.18

b) Transmission (Mn K edge)

Sample charge state	Coated *2+*	Coated *3+*	Coated *$LiMn_2O_4$*	Coated $Li_{0.09}Mn_2O_4$	Coated *4+*	Uncoated *2+*	Uncoated *3+*	Uncoated *$LiMn_2O_4$*	Uncoated $Li_{0.09}Mn_2O_4$	Uncoated *4+*
4.35 V	*0.5 ± 0.2*			*97 ± 1*	*2.5 ± 1*	*7 ± 3*			*76 ± 2*	*17 ± 2*
3 V			*100*				*9 ± 2*	*91 ± 1*		

c)

Sample charge state	Coated	Uncoated
4.35 V	3.95 ± 0.08	3.83 ± 0.20
3 V	3.5	3.46 ± 0.09

Table S1. Species concentration (%) and average Mn oxidation state of coated and uncoated LMO samples at two different cycle stages. (a) LCF of the TEY spectra at the L_{23} Mn edges. The references were MnO (2+), Mn_2O_3 (3+) and MnO_2 (4+); (b) LCF of the transmission measurements at the Mn K edge. 3+ is the $Li_2Mn_2O_4$ reference, 2+ is MnO and 4+ is Li_2MnO_3; (c) Average oxidation state obtained from the spectra acquired in transmission mode.

The LCF at Mn K edge of the coated sample at 3 V was carried out with the XANES spectra of $LiMn_2O_4$ reported by ref[2]. Among the LMO samples presented by the authors, characterized by different structural order, the XANES relative to the sample with the higher structural order was employed. The best LCF for the uncoated sample at 3 V (reduced chi-squared factor = 3.6×10^{-4}) was obtained including a component of tetragonal $Li_2Mn_2O_4$ [7] in agreement with the Jahn-Teller distortion effect

discussed in the main text. For the charged coated sample, the best fit (reduced chi-squared factor = 5.56 x 10^{-4}) was obtained with a reference of a delithiated LMO, for which the authors[9] estimated a 9% Li content with respect to the initial LMO pristine powder. Li_2MnO_3[3] and MnO[3] were included to the fit. The lower white line intensity of the uncoated sample correlates with the abundant presence of the two side product (chi-squared factor = 8.1 x 10^{-4}). In figure S1, LCF have been carried out on the uncoated samples, using the coated sample at 3 V as a reference for $LiMn_2O_4$ and the charged one as delithiated reference ($Li_xMn_2O_4$). It is shown how the inclusion of the XANES of the side products correlates with the reduction of the intensity of the white line.

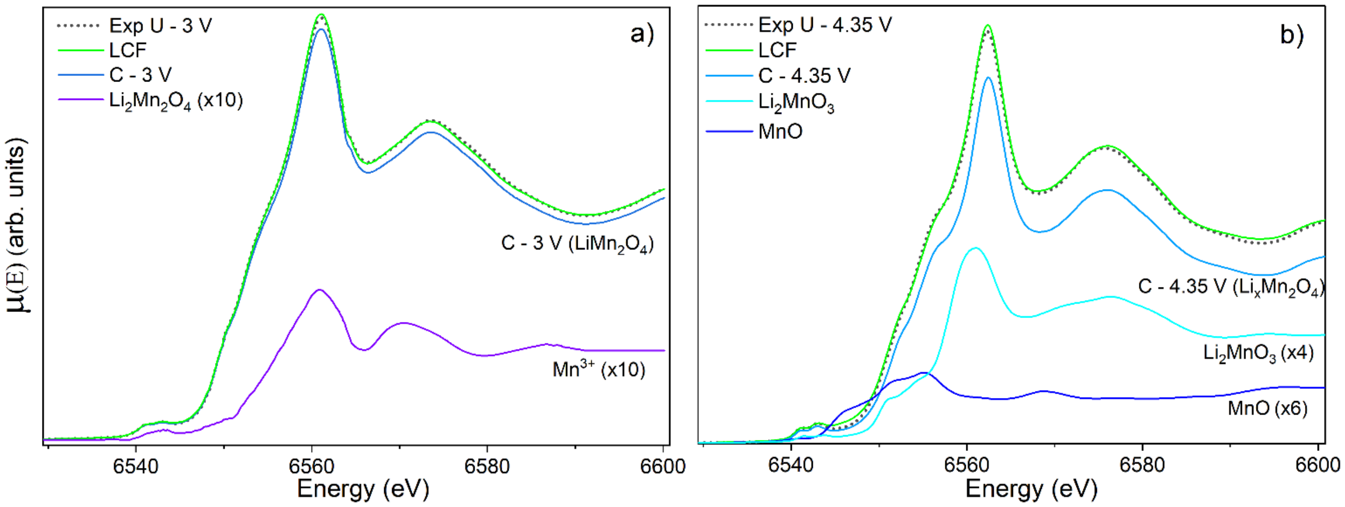

Figure S1. Linear Combination fit of the uncoated samples at 3 (a) and 4.35 V (b), using the coated samples as references.

Sample	C – 3 V	C – 4.35 V	MnO	$Li_2Mn_2O_4$	Li_2MnO3
**U – 3 V**	96.0 ± 0.4			4.0 ± 0.7	
**U – 4.35 V**		84.4 ± 0.7	3.2 ± 0.2		12.4 ± 0.7

Table S2. Species concentration (%) obtained via LCF of the uncoated sample. Fit were realized using the coated sample at the same potential as a reference, and including the expected side products.

2. EXAFS Analysis

The starting structure utilized for the EXAFS analysis at the Mn k-edge is the cubic spinel $LiMn_2O_4$ [5]. Sample C – 3 V was fitted (R = 1.09x10^{-6}) with excellent agreement with the reference. The best fit of the uncoated sample at 3 V was realized adding to the same structure the first Mn-O shell simulated from tetragonal $Li_2Mn_2O_4$ [7], obtaining an R factor of 1.13x10^{-6}. Fig. S2 (right) shows the lower Mn-Mn/Mn-O intensity ratio on the Fourier transform of the uncoated sample, as well as the decrease of the other scattering peaks intensity. This is in agreement with the formation of $Li_2Mn_2O_4$ [10].
Sample C – 4.35 V was again fitted from the same cubic LMO structure, obtaining a R factor of 3.66x10^{-6}. To improve the fit of the uncoated charged sample, the first shell of MnO[4] and Li_2MnO_3[8] were included into the fit (R factor = 3.84x10^{-6}), considering a tetrahedral coordination of the Mn atoms in MnO. The presence of these side products, as shown by Fig. S2 (left), is pinpointed by the broadening between the first two shells and the Mn-O intensity decrease.

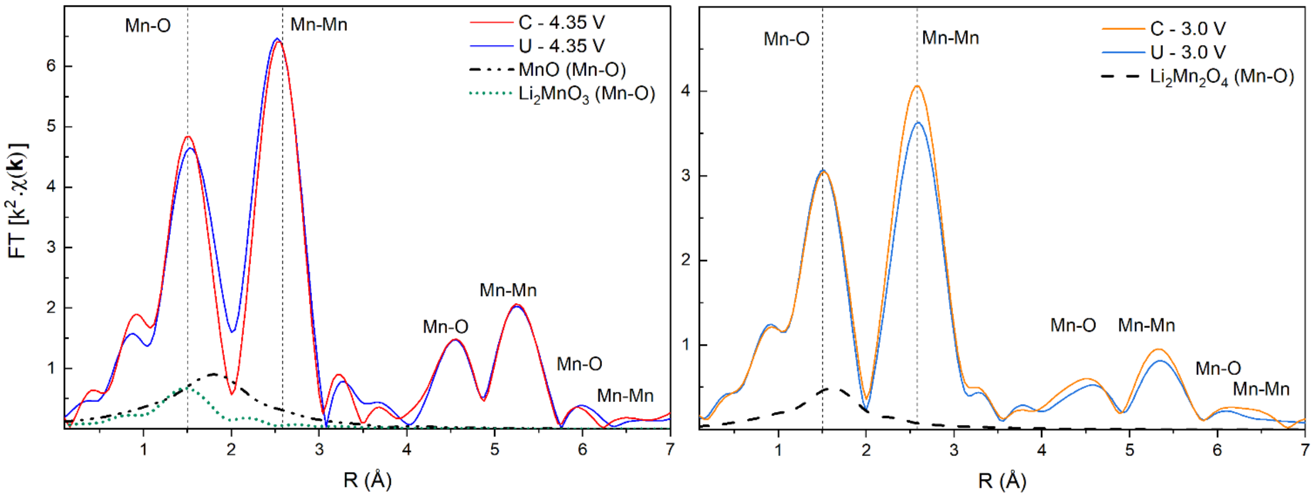

Figure S2. Comparison of the FT of the EXAFS spectra relative to coated and uncoated sample at 4.35 V (left) and 3 V (right). The side products signal included to the fit are shown.

3. Structure Diagrams

Li de-intercalation is expected to result in the formation of $LI_xMn_2O_4$. The complete Li extraction, when the distortions are limited and the cubic structure is preserved, result in the formation of a spinel-like MnO_2 structure, λ-MnO_2, that maintain the original symmetry.

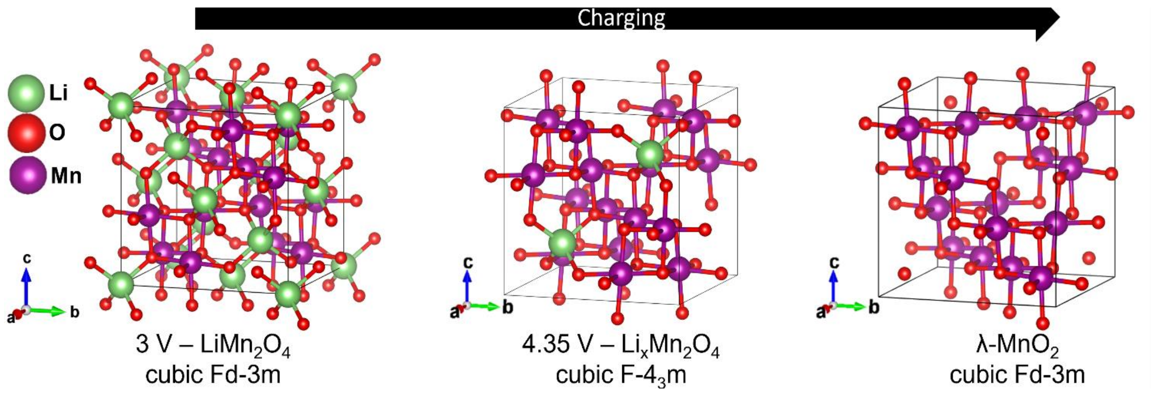

Figure S3. Diagram of the Li extraction during the charging process of spinel cubic LMO.

Bibliography

[1] S. J. Rezvani, R. Parmar, F. Maroni, F. Nobili, A. Di Cicco, and R. Gunnella, Does alumina coating alter the solid permeable interphase dynamics in $LiMn_2O_4$ cathodes?, The Journal of Physical Chemistry C 124, 26670 (2020).

[2] Jyh-Fu Lee, Y.-W. T.-H. (2003). Local structure transformation of nano-sized Al-doped $LiMn_2O_4$ sintered at different temperatures. *Journal of Power Sources*, 119-121:721-726.

[3] Johnson, C. S. et al., Structural Characterization of Layered $Li_xNi_{0.5}Mn_{0.5}O_2$ (0 < x < 2) Oxide Electrodes for Li Batteries. Chemistry of Materials (2003), 15, 2313–2322.

[4] The Materials Project. (10. 11 2021). Data retrieved from the Materials Project for MnO_2 (mp-25275) from database version : https://materialsproject.org/materials/mp25275?_sort_fields=symmetry.crystal_system&formula=MnO2

[5] The Materials Project. Materials Data on $LiMn_2O_4$ (SG:227) by Materials Project. United States. doi:https://doi.org/10.17188/1200415

[6] The Materials Project. (2020). Data on MnO by Materials Project. United States: N. p.,: doi:10.17188/1193794

[7] The Materials Project. (2021). Data on $LiMnO_2$ by Materials Project. United States.: doi:https://doi.org/10.17188/1207387

[8] The Materials Project. Data on Li_2MnO_3 by Materials Project. United States. doi:https://doi.org/10.17188/1193768.

[9] Youhei Shiraishi, I. N. (1997). In situ Transmission X-Ray Absorption Fine Structure Analysis of the Charge-Discharge process in $LiMn_2O_4$, a rechargeable Lithium Battery Material. *Journal of solid state chamistry, 133*, 587-590.

[10] S.-J. Hwang et al. (2002). Variation of chemical bonding nature of layered $LiMnO_2$ upon delithiation/relithiation and Cr substitution. Solid State Ionics 151 (2002) 275–283.

[11] B. Gilbert et al., Multiple scattering calculations of bonding and x-ray absorption spectroscopy of manganese oxides, The Journal of Physical Chemistry A 107, 2839 (2003).
